# Adherence to Recommended Breast Cancer Screening in Iranian Turkmen Women: The Role of Knowledge and Beliefs

**DOI:** 10.5402/2013/581027

**Published:** 2013-04-16

**Authors:** Abdurrahman Charkazi, Afieh Samimi, Khadijeh Razzaghi, Ghorban Mohammad Kouchaki, Mitra Moodi, Kamal Meirkarimi, Ashoor Mohammad Kouchaki, Hossein Shahnazi

**Affiliations:** ^1^Department of Public Health, School of Health, Golestan University of Medical Sciences, Km 2 Gorgan-Sari Road, Gorgan, Iran; ^2^School of Health, Golestan university of Medical Sciences, Gorgan, Iran; ^3^Department of Surgery, School of Paramedic, Golestan University of Medical Sciences, Gorgan, Iran; ^4^Department of Public Health, School of Health, Birjand University of Medical Sciences, Birjand, Iran; ^5^School of Health, Isfahan University of Medical Sciences, Isfahan, Iran; ^6^Department of Psychology, Islamic Azad University, Azadshahr Branch, Azadshahr, Iran; ^7^Department of Health Promotion and Health Education, School of Health, Isfahan University of Medical Sciences, Isfahan, Iran

## Abstract

The aim of the current study was to investigate breast cancer screening performance among Iranian Turkmen women along with their knowledge and beliefs. A cross-sectional study was carried out in June to December 2011. Through clustered sampling method, 1080 Iranian Turkmen women completed the questionnaire including breast cancer screening adherence, knowledge, fatalism beliefs, and perceived threat using Champions Health Belief Model Scale (CHBMS).The mean age of the participants was 43.04 (SD = 11.80) years. Compliance rate in a regular basis based on national guidelines was 13.1%, 2.5%, and 0.9% for SBE, CBE, and mammography, respectively. A mere 4% have been provided adequately with information about breast cancer. Having knowledge was the best predictor of breast cancer screening adherence along with high educated husbands for SBE performing. Susceptibility and fatalism were low and were influenced by participants' educational level and age. In conclusion, Iranian Turkmen women had insufficient knowledge, low perceived susceptibility, high fatalistic belief, and very poor adherence to breast cancer screening. There is a need for providing breast cancer education programs among the Iranian Turkmen women to increase their adherence rate.

## 1. Introduction

Breast cancer is the most common type of cancer among women and its incidence rates are increasing throughout the world [1, 2]. According to Iranian population-based cancer registration report, breast cancer is becoming number one prevalent cancer among Iranian women accounting for 24.82% of all cancers diagnosed among women. Furthermore, 6456 new cases of breast cancer were diagnosed in 2006 [3]. The age-adjusted incidence rate was 25.06 per 100,000 women in this period [3]. Harrichi et al. study acknowledged that Iranian women get to breast cancer at least one decade younger than women in developed countries [4]. 

Essential to the success of decreasing mortality, early detection and breast cancer diagnosis is logically a significant process and it could lead consequently to an increase in the survival rate up to 95% [1]. There has not been any systematic preventive approach in Iranian health system affecting the early detection and treatment of breast cancer among Iranian women. Specialists defined breast cancer screening behaviors as health improvement activities,  like breast self-examination (BSE), clinical breast examination (CBE), and mammography which facilitate early detection [5, 6]. Despite the effectiveness of breast cancer screening behaviors in reducing mortality, research findings indicate that screening rates remain low in Iranian women yet [7].

The Health Belief Model (HBM) has provided a useful conceptual framework to prevent health behaviors. According to HBM, threat perception and behavioral evolution are the two main components which are derived from expectancy-value theory. Threat perception included two key beliefs: perceived susceptibility and anticipated severity of contracting health conditions [8]. As identified from the HBM, individuals are more likely to engage in preventive health behaviors if they perceive themselves to be susceptible to a certain disease/illness (perceived susceptibility), perceive the condition to have potentially serious consequence (perceived severity), believe that a course of action will produce positive outcomes (perceived benefits), or perceive that obstacles or barriers to taking actions are outweighed by the benefits [9, 10]. 

The model states internal and external cues such as body states and environmental factors which may also promote or inhibit health enhancing behavior (cues to action), the confidence that one can successfully practice the behavior required to produce the outcome (self-efficacy), and the desire to maintain good health status (health motivation) were added to the model [11]. HBM has been widely used for recommended breast cancer screening compliance. In 1980s, Champion developed scales to early detection of breast cancer, based on the HBM [12, 13]. Champions Health Belief Model Scales (CHBMS) have been translated into several languages and tested in a number of cultures and populations [14–20]. 

Researchers have found that the fatalistic attitudes are the major barrier to recommended breast cancer screening uptake. Fatalism is identified as a doctrine of fate, a philosophical doctrine held by individuals who believe that all events are fated to happen and that human beings have no control over their futures and are unable to change their outcomes [21]. Fatalism is the belief that situations, including illnesses or catastrophic events, happen because of a higher power (such as God) or they are just meant to happen and cannot be avoided [22]. 

Turkmen people are a racial group that reside in northern and north east of the Islamic Republic of Iran and composed about 0.8% of the Iranian population. This population group has different culture, religion, and language from the most part of the country. There is not any survey that reported this racial minority with the topic of breast cancer screening behaviors. Studies indicated that along with demographic characteristics, the health belief variables such as perceived susceptibility to breast cancer, seriousness of breast cancer can influence the performance of BSE, CBE, and mammography [23–25]. Hence, current survey was aimed to assess the Turkmen women knowledge, perceived threat, fatalism belief, and practice to breast cancer screening behaviors.

## 2. Methods

### 2.1. Study Design

A cross-sectional study was carried out in June to December 2011, simultaneously in Gonbade Qabous and Turkmen districts in Golestan province where their main residents are Turkmen people. Prior to data collection, the study was approved by the Golestan University of Medical Sciences Authorities. Women gave verbal consent to participate after being given assurances of confidentiality and anonymity, and no incentives were offered for completing the survey. The inclusion criteria for participants were 30 years of age or older, women who are Turkmen ethnic group, and capable of communicating in Turkmen language. 

 The perceived susceptibility and perceived severity related to breast cancer instruments were translated using the Banville method, to develop a cultural adaptation [26]. In brief the original instruments were translated into Turkmen language by three students of Ph.D. and M. S. degrees in health education and five bilingual health professionals back translated the instruments without access to any original English version independently. These three versions then were compared, evaluated, and modified to reconcile any differences observed. Content validity suitable for the purpose of the study was established via asking for its quantified clarity and linguistic appropriateness from 4 Iranian experts in health education. Then, edited versions were given to a sample of 30 Turkmen women in pilot phase for its reliability. The reliability assessed by internal consistency with Cronbach's alpha was acceptable (*α* = 0.742).

To recruit eligible women to participate in this study, we had recourse to a number of 10 health centers in the cities and 60 rural health houses, providing and including all family records basis on primary health cares. 

### 2.2. Sample

Participants were a clustered randomized sample of 1080 Turkmen women. Their ages ranged between 30 and 82 years, with a mean age of 43.04 ± 11.80 years. The majority of them (85.3%) were married, 5.8% unmarried, and the remainder divorced or separated. Demographic profiles of the participants are reported in Table 1. 

### 2.3. Measures

The questionnaire consisted of four parts: (1) demographic variables included questions about age, level of education, current marital status, children's number, job, and breast cancer risk factors such as history of oral contraceptive use, family history of breast cancer, and whether breastfed their child/children; (2) knowledge-related breast cancer screening: hearing of BSE, CBE, and mammography, periodic time of BSE, CBE, and mammography, and receiving of first mammogram time; (3) practice for breast cancer screening behaviors: recommended frequency of BSE, CBE, mammography, and regular/irregular basis; (4) susceptibility (3 items) and seriousness (7 items) to breast cancer were assessed using the Champions Health Belief Model Scales (CHBMS) [13, 15]. Participants' rating was made on a 5-point Likert scale, ranging from “strongly agree” to “strongly disagree”. Due to Muslim society basis of the samples, the fatalistic belief role like getting breast cancer is the will of god or chance was assessed. Possible responses in susceptibility, seriousness, and fatalism ranged from one (strongly disagree) to five (strongly agree). Each subscale was scored by calculating the means of all item scores [1–5]. Breast cancer screening compliance was assessed by women's self-reports on BSE, CBE, and mammography.

### 2.4. Analysis

All data were entered in the SPSS (version 15) Windows software. Descriptive statistics were used in demographic characteristics and other variables. Chi-Square test was applied to on the difference between breast cancer screening performance and selected demographic variables. One-way analysis of variances and Mann-Whitney *U* tests were conducted for any differences between perceived threat and fatalism with demographic variables and BSE, CBE, and mammography performance. Logistic regression analysis was performed to identify the extent to which individual variables significantly predicted breast cancer screening behaviors. In all tests, values of *P* ≤ 0.05 were considered significant. 

## 3. Findings

### 3.1. Breast Cancer Knowledge

According to Table 2, only 4% reported that they have been provided adequately with information about breast cancer; meanwhile, 60.5% reported their knowledge in this area is not suitable. Friends (36.7%), mass media consisting of radio and TV (20.6%), and health professionals (10.9%) were the best source of the breast cancer information. The vast majority of the women (71.9%) were interested in receiving more information. From their point of view, 61.7% preferred to receive information by CD, printed material (8.3%), and telephone (1.5%), and the rest of them (28.5%) did not report any preferred method. 

When asked about their common barrier to participating in breast cancer educational session, they listed lack of time (47.8%), followed by transportation (20.7%), did not want to think about breast cancer (10.4%), and did not believe that breast cancer is important (2.5%), and 18.6% did not report any reasons for barriers.

When women were asked if they had heard something about BSE, CBE, and mammography, 42.2% reported that they heard about BSE, 32.7% about CBE, and 24.4% about mammography. Results regarding breast cancer screening behaviors based on national guidelines revealed that the majority of women (94.4%) did not know anything about monthly BSE. Only 1.9% correctly reported that CBE every three years should be performed between the ages of 20 and 39 years. The annual yearly mammography screening test started at the age of 40 was reported by 4.5% of the participants.

### 3.2. Performance: Adherence to Breast Cancer Screening Guidelines

Seventy-six point eight percent of the participants had not ever performed BSE, followed by 93% for CBE, and 97.8% for mammography. Last month performance of BSE among those who have 30 years and older was 13.1%. Among those who have 40 years and older, mammogram in the past year was .9% and CBE in the past year was 2.5%. When women were asked if they were acquainted with any person who had breast cancer, they reported only one person having breast cancer themselves, 0.8%, 0.7%, 0.1%, 10.7%, and 4.2% reported that their mother, sister, daughter, close relatives (except family members), and friends have breast cancer, respectively. 42.5% delineated they have also lie with breast cancer.

The breast cancer screening behaviors were influenced by age group except for mammography (Table 2) and were not influenced by educational level except for CBE (Table 3). Significant differences were observed between exposure to breast cancer (*χ* ^2^ = 16.208, *P* = 0.000) and current marital status (*χ* ^2^ = 4.265, *P* = 0.39) with performance of BSE. Fisher's Exact test results showed current marital status and exposure to breast cancer were not influencing variables with performing CBE and mammogram. 

### 3.3. Perceived Threat and Fatalism

The results described that the mean and SD of susceptibility of the women were 2.82 ± 1.22, and this rate for their seriousness was 3.72 ± 0.7. The mean and SD of fatalism beliefs toward breast cancer were 3.86 ± 0.78. The ANOVA test showed there was a significant difference between susceptibility and fatalism with educational level (Table 4) but not significant with seriousness. Moreover, beliefs were influenced also by age group except for seriousness (Table 5). 

The Mann-Whitney *U* test showed significant differences between SBE performance and susceptibility (*U* = 57511, *P* = 0.007), but no significant differences emerged between the SBE practice with subscales of severity (*U* = 62277, *P* > 0.05) and fatalism (*U* = 62868.500, *P* > 0.05). This result repeated regarding CBE performance with the subscales of susceptibility (*U* = 2504.500, *P* = 0.026), severity (*U* = 3501, *P* > 0.05), and fatalism (*U* = 3165.500, *P* > 0.05) and mammogram practice with the subscales of susceptibility (*U* = 602.500, *P* = 0.027), severity (*U* = 905.500, *P* > 0.05), and fatalism (*U* = 974, *P* > 0.05).

Significant association was founded between exposure to the concept of perceived susceptibility (*U* = 116126, *P* = 0.000) and fatalistic belief (*U* = 125175.5, *P* = 0.001), but seriousness was not impressed by exposure to breast cancer (*U* = 137119.500, *P* > 0.05).

Logistic regression results showed that women's knowledge rate about breast cancer screening behaviors was the best predictor of BSE, CBE, and mammography adherence. Further, the husband education level was an another predictor of BSE performance (Table 6).

## 4. Discussion

Early detection of breast cancer via adherence to current screening behaviors is a key epigram to effective treatment and prognosis of this malignancy which can reduce morbidity and mortality. In this regard, the adherence rates may be influenced by some personal and sociodemographic variables as well as the individuals' knowledge and beliefs. The objective of the current study was to determine the SBE, CBE, and mammography performance in a sample of Iranian Turkmen women.

 The results of the study indicated the participants' knowledge and compliance with recommended screening practice is insufficient and poor. In this regard, the lack of sufficient knowledge of breast cancer reported by subjects may have influenced their low screening adherence. According to the knowledge, 96% said that their information related to breast cancer is insufficient and 71.9% were willing to receive personal education and would be willing to participate in this area by educational materials. This worthy intention for education is a good opportunity for local health care providers to implementing appropriate interventions for the enhancing breast cancer knowledge, belief, and screening compliance. In this regard, the time and transportation were the most reported barriers to participation in educational sessions, suggesting that to implementing of the interventions in women routine patterns of living and improving local transportation accessibility.

In the current study, a mere 13.1% of participants have had BSE during the last month and the vast majority of the participants (97.5%) at the age of 40 and older did not report CBE in the past year; this rate was 99.1% for mammogram. Our findings have been identical nearly to the results of other previous studies conducted in Iran, where breast cancer screening performance in accordance with national guidelines ranged from 4.5% to 42.7% for SBE, 3% to 29.5% for CBE, and 3.3% to 30.5% for mammography in different samples and varied ethnic groups [27–38]. Avci found that only 4.3% of female workers in Turkey sample performed SBE monthly basis [25]. SBE performance is very high in the sample of western and developed countries. Sadler et al. reported 30.9% of American Korean women having had BSE in regular frequency [39]. 

The results demonstrated that SBE performance was impressed by age, marital status, and exposure to breast cancer. Inconsistent with our finding Avci reported marital status and familial breast cancer history were significantly associated with BSE performance, but not associated by educational levels and age [25]. Jarvandi et al. study performing doing BSE was more frequent in married and exposed Iranian teachers, but contrary to our findings BSE performs higher in older women [40]. However, the low education level of participants should be considered. Contrary to our findings Petro-Nustus and Mikhail in a sample of Jordan women reported that the BSE was influenced by age and educational level [24]. This finding is lower than other surveys conducted in the country.

In line with mammography among women of 40 years and older, the very poor performance was observed. Iranian Ministry of Health recommended mammogram every 1-2 year at age of 40 and thereafter. The finding of the current study related to mammogram practice is lower than other parts of Iran. In contrast, mammography practice is higher in western countries, where Wu et al. reported that compliance with recommended screening practice is 64% among Filipino, Chinese, and Asian-Indian women of ethnic group in the United States [10]. Secginli's and Nahcivan's survey indicated that 25% of the Turkish women had received a mammogram within the past 2 years [41]. In Iran breast cancer screening is not the same as a routine health promoting programs in health sectors and it completely depends on physicians' recommendation and their referring to mammogram clinics, partially expensive self-financed and inadequate insurance coverage procedure. In this regard, the most common barriers of mammography such as time and transportation limits, insufficient coverage of insurance, low knowledge and susceptibility, and high fatalistic beliefs should be eliminated. Mobile mammogram van facilities, providing of breast cancer screening behaviors as a part of primary health care services, awareness and perceived risk increase, and women reminder systems like phone calls could be convincing women to schedule their mammography performance. 

Mammography adherence was not significantly associated with demographic characteristics like age, education, marital status, and breast cancer family. Contrary to our findings other studies demonstrated that annual mammogram performance varied according to age, educational level, marital status, and having breast cancer exposure [39, 42]. However, in this area the very low compliance of mammography should be considered. 

On the basis of the selected HBM subscales, only concept of susceptibility was positively associated with BSE, CBE, and mammography compliance. Women who performed BSE, CBE, and mammography perceived higher risk to breast cancer than those who did not practice BSE, CBE, and mammography. The concept of perceived severity is not significantly associated with screening behavior that is inconsistent with HBM theory. Our findings are in agreement and supported other studies, where they concluded that perceived severity may be that the breast cancer is regarded as a serious condition by most women [13, 25, 41, 42]. In this study performing BSE, CBE, and mammography was influenced by the concept of susceptibility, whereas women who had higher perceived susceptibility had greater compliance. The results of a meta-analytic review indicated that perceived breast cancer risk influences the adherence to mammography screening guidelines and is not clearly influencing adherence to breast self-examination [43]. In another study susceptibility perception of women who had BSE and mammogram was significantly higher than groups who had not BSE and mammogram [44].

In the current study, the education level and age were significantly associated with susceptibility; the younger and high educated women perceived higher risk to breast cancer. Meanwhile, the education level negatively correlated with seriousness and fatalism that poorly educated women are more likely to consider the breast cancer as severe condition and that its susceptibility is the will of God and depend on luck. In consistence with our findings, Katapodi et al. meta-analytic review demonstrated that perceived risk is weakly influenced by age and education. Seven of those studies concluded that younger women were more likely to perceive higher risk for developing breast cancer than older women [43]. The perceived susceptibility and fatalistic belief were influenced by exposure to breast cancer. In agreement with our survey, Finnery Rutten and Iannotti's study as well as Katapodi et al. study showed women who have their close relatives having breast cancer reported higher perceived risk than those who did not have exposure to breast cancer [43, 45]. However, having close relative/relatives could act as a cue to action that women perceived them in a greater and higher risk to breast cancer.

We found that fatalism belief was higher than perceived susceptibility and seriousness which could considered as a barrier to breast cancer screening behaviors and early detection. Fatalism belief had a positive significance with age; the older women had higher perceived fatalistic belief. This result supports previous findings suggesting that fatalistic belief is an important belief in Muslim societies [25, 46, 47]. Talbert in her study reported that fatalism belief scores were statistically related to breast cancer compliance among African American middle class women, as a psychosocial barrier that decreases screening compliance [47]. In this regard, providing the main causes of the breast cancer and important role of screening compliance by health providers, media, and other channels could be useful.

 In conclusion, the results of the current study revealed Turkmen women had poor knowledge, low perceived susceptibility, high fatalistic belief, and low adherence to breast cancer screening. For increasing breast cancer screening adherence of Turkmen women, conducting a holistic and comprehensive intervention through viable strategy is imperative.

### 4.1. Study Limitation

First, the study used a cross-sectional sample. Second, the self-report nature of questionnaire in spite of the anonymous nature of the questionnaires should be mentioned. Third, women's self-report in compliance with CBE and mammography screening was not validated with their medical records. Another limitation was that the concepts of perceived barriers, perceived benefits, self-efficacy, and health motivation were not survived. Thus, further studies are needed to examine these concepts in other geographic locations of Turkmen population women. In spite of this limitation, the current study demonstrated new sights for the understanding of perceived threat, fatalism, and behaviors of breast cancer screening in Iranian Turkmen women.

## Figures and Tables

**Table 1:** Demographic profile of the surveyed population.

Characteristics	*n* (%)
Age (*n* = 1080)	
30–39	522 (48.3)
40–49	249 (23.1)
50–59	177 (16.4)
≥60	132 (12.2)
Marital status (*n* = 1080)	
Married	921 (85.3)
Unmarried	63 (5.8)
Widowed	93 (8.6)
Divorced	3 (0.3)
Educational level (*n* = 1080)	
Illiterate	399 (37)
Less than 5 years	355 (33)
6–8 years	132 (12.1)
9–11 years	41 (3.8)
High school graduate	104 (9.6)
College level	49 (4.5)
Job	
Work in home	1030 (96)
Out of home	50 (4)
Breastfeeding experience	
Exclusive breastfeeding	776 (70.8)
Formula	48 (4.4)
Both of them	173 (16)
Unknown	94 (7.8)
Oral contraceptive use	
Yes	578 (53.5)
No	502 (46.5)

**Table 2:** Age groups and breast cancer screening behaviors in a sample of Iranian Turkmen women.

Age group	Performed BSE^a^ (within last month)			Performed CBE^b^ (within last year)			Performed mammogram^b^ (within last year)		
	Yes % (*n*)	No % (*n*)	Statistics	Yes % (*n*)	No % (*n*)	Statistics	Yes % (*n*)	No % (*n*)	Statistics
30–39	83 (15.9)	439 (84.1)	*χ* ^2^ = 12.264 df = 3, *P* = 0.007	32 (6.1)	490 (93.9)	*χ* ^2^ = 10.207 df = 2, *P* = 0.006	4 (0.8)	518 (99.2)	*χ* ^2^ = 2.490 df = 2, *P* = 0.288
40–49	36 (14.5)	213 (85.5)		12 (4.8)	237 (95.9)		2 (0.8)	247 (99.3)	
50–59	19 (10.7)	158 (89.3)		2 (1.1)	175 (98.9)		3 (1.7)	174 (98.3)	
≥60	6 (4.5)	126 (95.5)		0 (0)	132 (100)		0 (0)	132 (100)	

Total	144 (13.3)	936 (86.6)		46 (4.3)	1034 (95.7)		9 (0.8)	1071 (92.2)	

^a^Women of 30 years of age and older.

^b^Women of 40 years of age and older.

**Table 3:** Educational levels and breast cancer screening behaviors in a sample of Iranian Turkmen women.

Educational level	Performed BSE^a^ (within last month)			Performed CBE^b^ (within last year)			Performed mammogram^b^ (within last year)		
	Yes % (*n*)	No % (*n*)	Statistics	Yes % (*n*)	No % (*n*)	Statistics	Yes % (*n*)	No % (*n*)	Statistics
Illiterate	44 (11)	355 (89)	*χ* ^2^ = 5.794 df = 5, *P* = 0.327	12 (3)	387 (97)	*χ* ^2^ = 22.353 df = 5, *P* = 0.000	5 (1.3)	394 (98.7)	*χ* ^2^ = 4.835 df = 5, *P* = 0.436
Less than 5 years	47 (13.2)	308 (86.8)		11 (3.1)	344 (94.9)		2 (0.6)	353 (99.3)	
6–8 years	16 (12.1)	116 (87.9)		2 (1.5)	130 (98.5)		0 (0)	132 (100)	
9–11 years	4 (9.8)	37 (90.2)		1 (2.4)	40 (97.6)		0 (0)	41 (100)	
High school graduate	21 (20.2)	83 (79.8)		8 (7.7)	96 (92.3)		2 (1.9)	102 (92.2)	
College level	10 (20.4)	39 (89.6)		7 (14.3)	42 (85.7)		0 (0)	49 (100)	

Total	142 (13.2)	126 (86.8)		41 (3.8)	1039 (96.2)		9 (0.8)	1071 (99.2)	

^a^Women of 30 years of age and older.

^b^Women of 40 years of age and older.

**Table 4:** Beliefs related to breast cancer screening adherence and educational levels in a sample of Iranian Turkmen women.

Beliefs	Educational levels						*F*	Tukey's HSD^a^
	A (*n* = 397)	B (*n* = 352)	C (*n* = 131)	D (*n* = 41)	E (*n* = 104)	F (*n* = 49)
Susceptibility	2.66 ± 1.11	2.91 ± 1.20	2.78 ± .95	2.69 ± .96	3.07 ± 1.10	3.25 ± .95	4.805	A, B < C, D, E, F C, D, E < F
Seriousness	3.74 ± .73	3.82 ± .66	3.66 ± .67	3.65 ± .59	3.55 ± .75	3.57 ± .73	3.397	N.S
Fatalism	4.02 ± .68	3.96 ± .76	3.79 ± .69	3.45 ± .94	3.49 ± .89	3.26 ± .87	18.650	F, E, D < C, B, A, C < B, A

A: illiterate; B: less than 5 years; C: 6–8 years; D: 9–11 years; E: high school graduate; F: college level.

^a^Mean differences for the Tukey's HSD pairwise comparisons (*P* < .05).

**Table 5:** Beliefs related to breast cancer screening adherence and age groups in a sample of Iranian Turkmen women.

Beliefs	Age group				*F*	Tukey's HSD^a^
	A (*n* = 522)	B (*n* = 249)	C (*n* = 177)	D (*n* = 132)
Susceptibility	2.90 ± 1.10	2.89 ± 1.12	2.67 ± 1.16	2.57 ± 1.08	4.851	A, B > C, D
Seriousness	3.73 ± .67	3.79 ± .68	3.65 ± .79	3.67 ± .73	1.601	N.S
Fatalism	3.76 ± .81	3.93 ± .76	3.94 ± .77	4.03 ± .66	6.176	A < B, C, D B, C < D

A: 30–39 years; B: 40–49 years; C: 50–59 years; D: ≥60 years.

^a^Mean differences for the Tukey's HSD pairwise comparisons (*P* < .05).

**Table 6:** Logistic regression analysis for breast cancer screening performance among Turkmen women.

		*B*	SE	Wald	*P*	Exp(B)	95% CI	
							Lower	Upper
SBE	Knowledge	0.464	0.143	10.482	0.001	1.591	1.201	2.107
	Husband education	0.137	0.061	4.991	0.025	1.147	1.017	1.294
CBE	Knowledge	0.841	0.164	26.277	0.001	2.318	1.681	3.197
Mammography	Knowledge	0.995	0.261	14.559	0.001	2.704	1.626	4.507

## References

[1] American Cancer Society (2011). *Cancer Facts & Figures 2011*.

[2] Parkin DM, Fernández LMG (2006). Use of statistics to assess the global burden of breast cancer. *Breast Journal*.

[3] Iranian Ministry of Health and Medical Education (2008). *Iranian Annual of National Cancer Registration Report 2006–2007*.

[4] Harrichi I, Karbakhsh M, Kashefi A (2004). Breast cancer in Iran: results of a multi-center study. *Asian Pacific Journal Cancer Prevvention*.

[5] Yarbrough SS, Braden CJ (2001). Utility of health belief model as a guide for explaining or predicting breast cancer screening behaviours. *Journal of Advanced Nursing*.

[6] Lu ZYJ (2001). Effectiveness of breast self-examination nursing interventions for Taiwanese community target groups. *Journal of Advanced Nursing*.

[7] Noroozi A, Jomand T, Tahmasebi R (2011). Determinants of breast self-examination performance among iranian women: an application of the health belief model. *Journal of Cancer Education*.

[8] Clark NM, Becker MH, Shumaker S, Schron EB, Ockene JK, McBee WL (1998). Theoretical models and strategies for improving adherence and disease management. *The Handbook of Health Behavior Change: Second Edition*.

[9] Wu TY, Bancroft J, Guthrie B (2005). An integrative review on breast cancer screening practice and correlates among Chinese, Korean, Filipino, asnd Asian Indian American women. *Health Care for Women International*.

[10] Wu TY, West B, Chen YW, Hergert C (2006). Health beliefs and practices related to breast cancer screening in Filipino, Chinese and Asian-Indian women. *Cancer Detection and Prevention*.

[11] Harrison JA, Mullen PD, Green LW (1992). A meta-analysis of studies of the health belief model with adults. *Health Education Research*.

[12] Champion VL (1999). Revised susceptibility, benefits, and barriers scale for mammography screening. *Resarch Nursing Health*.

[13] Champion VL (1993). Instrument refinement for breast cancer screening behaviors. *Nursing Research*.

[14] Lee EH, Kim JS, Song MS (2002). Translation and validation of Champion’s Health Belief Model Scale with Korean women. *Cancer Nursing*.

[15] Champion VL, Scott CR (1997). Reliability and validity of breast cancer screening belief scales in African American women. *Nursing Research*.

[16] Parsa P, Kandiah M, Mohd Nasir MT, Hejar AR, Nor Afiah MZ (2008). Reliability and validity of Champion’s Health Belief Model Scale for breast cancer screening among Malaysian women. *Singapore Medical Journal*.

[17] Secginli S, Nahcivan NO (2004). Reliability and validity of the breast cancer screening belief scale among Turkish women. *Cancer Nursing*.

[18] Mikhail BI, Petro-Nustas WI (2001). Transcultural adaptation of Champion’s health belief model scales. *Journal of Nursing Scholar*.

[19] Wu TY, Yu MY (2003). Reliability and validity of the mammography screening beliefs questionnaire among Chinese American women. *Cancer Nursing*.

[20] Taymoori P, Berry T (2009). The validity and reliability of champion’s health belief model scale for breast cancer screening behaviors among Iranian women. *Cancer Nursing*.

[21] Franklin MD, Schlundt DG, McClellan LH (2007). Religious fatalism and its association with health behaviors and outcomes. *The American Journal of Health Behavior*.

[22] Austin LT, Ahmad F, McNally MJ, Stewart DE (2002). Breast and cervical cancer screening in Hispanic women: a literature review using the health belief model. *Women’s Health Issues*.

[23] Juon HS, Seo YJ, Kim MT (2002). Breast and cervical cancer screening among Korean American elderly women. *European Journal of Oncology Nursing*.

[24] Petro-Nustus W, Mikhail BI (2002). Factors associated with breast self-examination among Jordanian women. *Public Health Nursing*.

[25] Avci IA (2008). Factors associated with breast self-examination practices and beliefs in female workers at a Muslim community. *European Journal of Oncology Nursing*.

[26] Banville D, Desrosiers P, Genet-Volet Y (2000). Translating questionnaires and inventories using a cross-cultural translation technique. *Journal of Teaching in Physical Education*.

[27] Hadizadeh F (2005). The effect of a training curriculum on attitude of female students about breast self-examination by using health belief model (HBM). *Journal of Birjand University of Medical Sciences*.

[28] Moodi M, Mood MB, Sharifirad GR, Shahnazi H, Sharifzadeh G (2011). Evaluation of breast self-examination program using health belief model in female students. *Journal of Research in Medical Sciences*.

[29] Abedian Kasgari K, Yagoubi T T (2011). Health believes of women about performing mammography among clients referred to health centers in Sari, Iran. *Journal of Mazanderan University of Medical Sciences*.

[30] Farshbaf Khalili A, Ghahvechi A, Thorabi SH (2009). Performance conditions of breast cancer screening methods and its efficient factors among women referring to health centers of Tabriz. *Iranian Journal of Nursing Research*.

[31] Karimi H (2005). Effect of breast self-examination (BSE) education on increasing women’s knowledge and practice, Ramsar. *Journal of Babol University of Medical Sciences*.

[32] Banaeian SH, Kheiri S S (2006). Knowledge, attitude and practice about breast cancer screening and related factors among women referred to health care centers in Boroujen in 2005. *Journal of Shahrekord University of Medical Sciences*.

[33] Mojahed SH, Dafei M (2001). Nursing-midwifery BSE knowledge and practice in Yazd. *Journal of Yazd University of Medical Sciences*.

[34] Godazandeh GH, Khalilian AR, Atarod Z (2006). Knowledge and practice of above15years old females towards breast cancer prevention in Sari township, 2004. *Journal of Mazandaran University of Medical Sciences*.

[35] Enjezab BF, Bokaei M (2004). Barriers and motivators related to cervical and breast cancer screening. *Journal of Yazd University of Medical Sciences*.

[36] Gholizadeh L, Mohamadhoseini S (2008). Barriers related to clinical breast examination in women whoreferred to health and medical centers of Gachsaran. *Journal of Yasuj University of Medical Sciences*.

[37] Alavi GH, Fattahi-Masoom AS, Shakeri MT (2010). Evaluation of prevalence of cervical and breast cancer screening programs between gynecologists. *Iranian Journal of Obstetrics, Gynecology and Infertility*.

[38] Soltanahmadi AA, Tirgari B (2010). A survey on the rate and causes of women’s participation or nonparticipation in breast and cervical cancers screening programs. *Iranian Journal of Obstetrics, Gynecology and Infertility*.

[39] Sadler GR, Ko CM, Cohn JA, White M, Weldon RN, Wu P (2007). Breast cancer knowledge, attitudes, and screening behaviors among African American women: the Black cosmetologists promoting health program. *BMC Public Health*.

[40] Jarvandi S, Montazeri A, Harirchi I, Kazemnejad A (2002). Beliefs and behaviours of Iranian teachers toward early detection of breast cancer and breast self-examination. *Public Health*.

[41] Secginli S, Nahcivan NO (2006). Factors associated with breast cancer screening behaviours in a sample of Turkish women: a questionnaire survey. *International Journal of Nursing Studies*.

[42] Fung SY (1998). Factors associated with breast self-examination behaviour among Chinese women in Hong Kong. *Patient Education and Counseling*.

[43] Katapodi MC, Lee KA, Facione NC, Dodd MJ (2004). Predictors of perceived breast cancer risk and the relation between perceived risk and breast cancer screening: a meta-analytic review. *Preventive Medicine*.

[44] Canbulat N, Uzun Ö (2008). Health beliefs and breast cancer screening behaviors among female health workers in Turkey. *European Journal of Oncology Nursing*.

[45] Finney Rutten LJ, Iannotti RJ (2003). Health beliefs, salience of breast cancer family history, and involvement with breast cancer issues: adherence to annual mammography screening recommendations. *Cancer Detection and Prevention*.

[46] Halligan P (2006). Caring for patients of Islamic denomination: critical care nurses’ experiences in Saudi Arabia. *Journal of Clinical Nursing*.

[47] Talbert PY (2008). The Relationship of fear and fatalism with breast cancer screening among a selected target population of African American middle class women. *Journal of Social Behavior and Health Sciences*.

